# Coupled Néel domain wall motion in sandwiched perpendicular magnetic anisotropy nanowires

**DOI:** 10.1038/srep08754

**Published:** 2015-03-04

**Authors:** I. Purnama, I. S. Kerk, G. J. Lim, W. S. Lew

**Affiliations:** 1 School of Physical and Mathematical Sciences, Nanyang Technological University, 21 Nanyang Link, Singapore 637371

## Abstract

The operating performance of a domain wall-based magnetic device relies on the controlled motion of the domain walls within the ferromagnetic nanowires. Here, we report on the dynamics of coupled Néel domain wall in perpendicular magnetic anisotropy (PMA) nanowires via micromagnetic simulations. The coupled Néel domain wall is obtained in a sandwich structure, where two PMA nanowires that are separated by an insulating layer are stacked vertically. Under the application of high current density, we found that the Walker breakdown phenomenon is suppressed in the sandwich structure. Consequently, the coupled Néel domain wall of the sandwich structure is able to move faster as compared to individual domain walls in a single PMA nanowire.

One of the key factors that determines the performance of magnetic domain wall (DW)-based devices^1^,^2^ is the ability to drive the DWs as fast as possible upon the application of current. Initially, materials such as permalloy (Ni_80_Fe_20_) with in-plane magnetic anisotropy are considered, as these materials possess high anisotropy magnetoresistance for the ease of DW detection^3^. Moreover, the DWs in in-plane materials are forced to rotate along the in-plane orientation, which is also known as the Néel configuration. DWs in in-plane materials have been shown to be able to move with a high speed under the application of current^4^,^5^. However, it has also been shown that the motions of DWs within in-plane materials are often not reproducible^6^. Materials with perpendicular magnetic anisotropy (PMA) have since been considered as an alternative for DW-based devices. As compared to the in-plane material, a DW in a single PMA nanowire under zero applied current typically adopts the Bloch configuration^7^, where the magnetization within the DW rotates out-of-plane. When high current density is applied to drive the DW, the magnetization within the DW is tilted continuously, which causes the DW shape to change periodically. This phenomenon is also known as the Walker breakdown, where the DW continuously switches between Bloch and Néel configurations, as shown in Fig. 1 (a)^7^,^8^. Recently, it has been shown that the storage density of a PMA nanowire can be increased by placing the nanowire on top of a granular layer to create a coupled-granular-continuous structure (CGC)^9^,^10^. The interlayer coupling between the granular layer and the PMA nanowire results in the decrease in the DW width which further results in the increase of the storage density.

In this work, we show by micromagnetic simulations that in a sandwich structure, where two PMA nanowires that are separated by a non-magnetic insulating spacer layer are stacked vertically, the DWs are coupled to each other in the Néel configurations. The sandwich structure differs from the CGC structure because here there exists a DW in both nanowires. Due to the coupling, it is possible to move the coupled DWs by only applying current to one of nanowires. Furthermore, the Walker breakdown phenomenon is observed to be suppressed. Under equal applied current density, the coupled DWs from the sandwich structure are able to move faster than individual DWs from a single PMA nanowire. By analysing the magnetization configurations of the sandwich structure, we found that the Walker breakdown suppression can be attributed to the curled magnetic domains. We have also investigated the dynamics of the coupled DWs in a sandwich structure with three PMA nanowires. In the three nanowire system, the magnetic domain in the middle nanowire is uncurled, making the coupled DW dynamics of the three-nanowire system to be similar to that of a single nanowire system.

Fig. 1 (b) shows the dynamics of a single DW in a single PMA nanowire under applied current density of *J* = 1.6 × 10^12^ A/m^2^. The simulation shows that the DW moves linearly under the application of current, and the motion is followed by a continuous rotation by the DW magnetization within the *xy* plane. The single DW is at the Bloch state when its magnetization is aligned along the *x* axis (*M_y_* = 0), and it is at the Néel state when the magnetization is aligned along the *y* axis (*M_y_* = ±1). Due to the width of the nanowire (*w* = 40 nm, length 5 μm), the energy difference between the Bloch state and the Néel state is close to zero^11^,^17^, which explains why the single DW appears to move linearly even when the applied current density is already in the Walker breakdown regime.

## Results

### Coupled Domain Wall Dynamics in Two-Nanowire System

**Figure 1:**
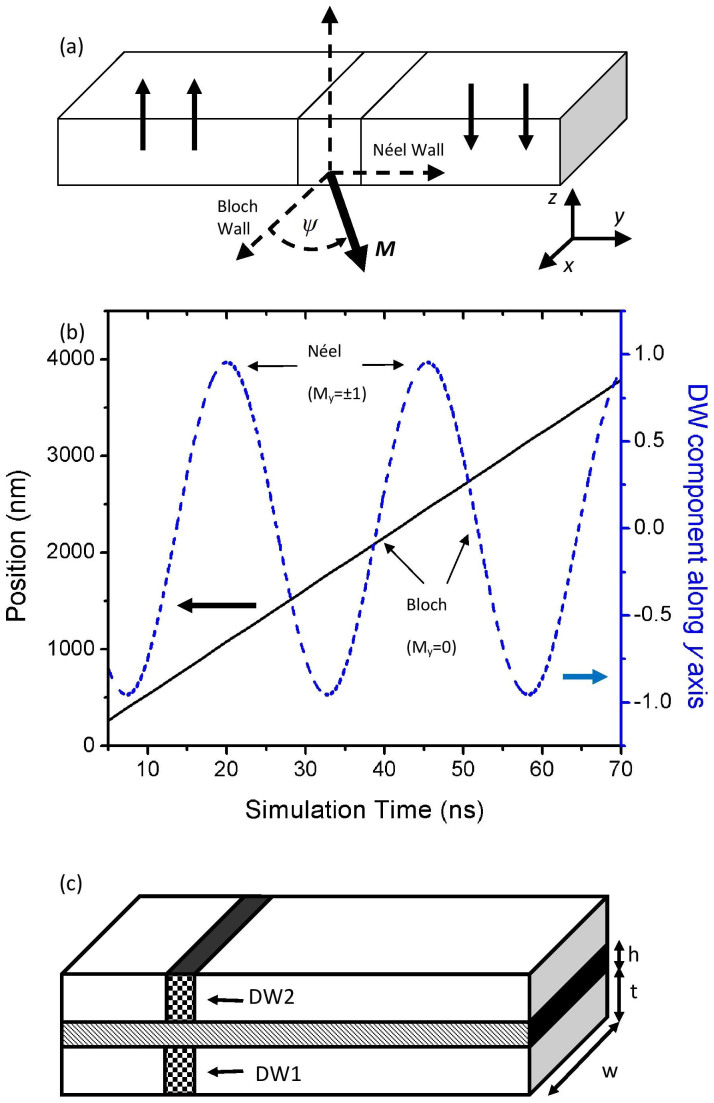
Dynamics of one PMA DW, followed by the schematic of the two-nanowire system. (a) Schematic of a Bloch DW in a single PMA nanowire. The DW magnetization is tilted by *ψ* in the *xy* plane when the DW is driven by current. (b) Plot showing the position of a current-driven DW together with the change in the DW magnetization component (*y* axis) as functions of time. (c) Schematic of the simulation model that is employed to investigate the DW dynamics in a sandwich PMA structure.

**Figure 2:**
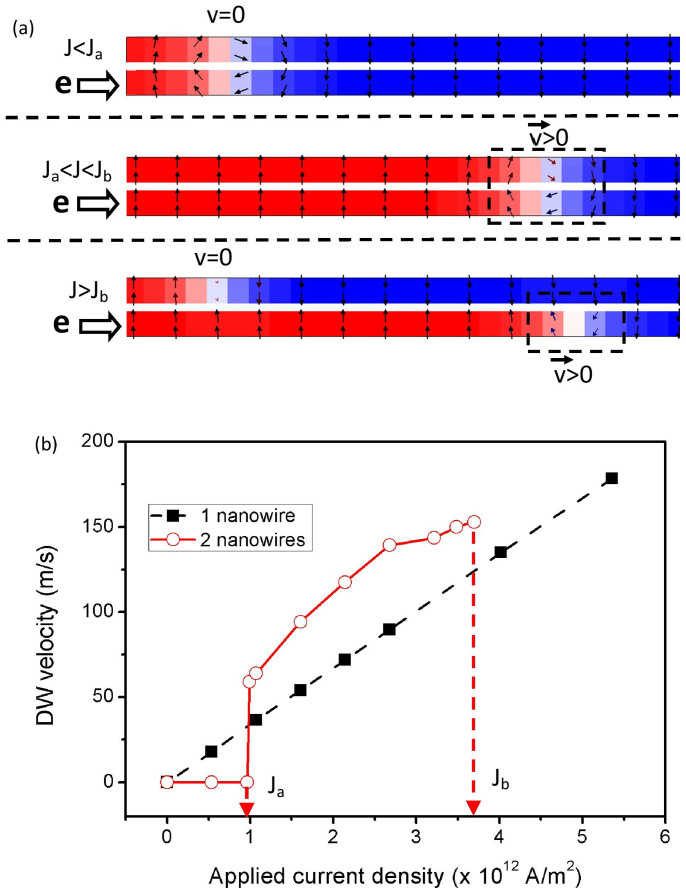
Dynamics of two coupled-PMA DWs of the two-nanowire system. (a) Snapshots of the two-nanowire simulations for various range of applied current density (side-view). Current is applied to the bottom nanowire. (b) Plot showing the relation between the speed of the coupled DWs and the applied current density. The speed of a single DW is included as comparison.

First, the dynamics of two DWs in a system of two PMA nanowires that are stacked in a sandwich structure are investigated. Fig. 1 (c) shows the schematic diagram of the simulation model, whereby two PMA nanowires were stacked vertically with a non-magnetic insulating spacer layer between them (See Supplementary Information section SI-5)^18^. Initially, a DW is generated at each nanowire at the same position with respect to the *y* axis. Spin-polarized current is then applied to the bottom PMA nanowire with the intent to drive the bottom DW only. Fig. 2 (a) shows the snapshots of the simulations (side-view) for different applied current density. The DWs in the sandwich structure are found to behave differently depending on the applied current density. For low applied current density, the bottom DW is pinned directly below the upper DW. This implies that a local pinning site is induced within the bottom nanowire and vice versa. The bottom DW remains stationary (pinned at the initial position) until the applied current density is increased to *J_a_* = 0.96 × 10^12^ A/m^2^, where it starts to propagate along the bottom PMA nanowire. However, the upper DW is also shown to be driven at the same instance in the same direction and speed as the bottom DW, even though there is no current applied to the upper PMA nanowire. The remote-driving of the upper DW in the absence of direct current shows that the two DWs are coupled to each other^12^,^13^,^18^. The relation between the speed of the coupled DWs of the two-nanowire system and the applied current is shown in Fig. 2 (b), the speed plot of a single DW is also included for comparison. As compared to the single nanowire system, the coupled DWs of the sandwich structure move with a noticeably higher speed. For instance, with applied current density of *J* = 2.68 × 10^12^ A/m^2^, the coupled DWs move with a speed of ≈140 m/s, which is approximately 50% faster than the single DW. Increasing the applied current density beyond *J_b_* = 3.7 × 10^12^ A/m^2^ results in the breaking of the coupling, which allows the bottom DW to move independently from the upper DW. Similar results are obtained when current is applied to the upper nanowire.

**Figure 3:**
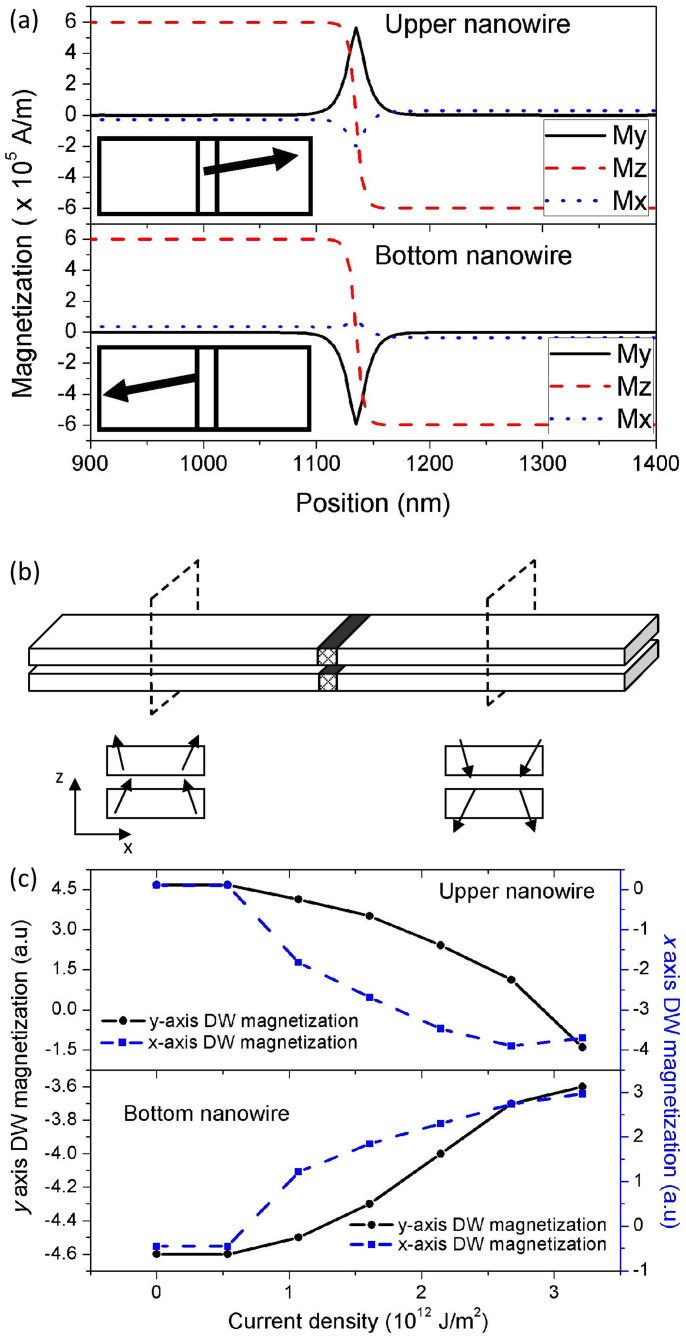
Magnetization configuration of the two-nanowire system. (a) The magnetization configuration of the two-nanowire system (left-edge of nanowire, x = 5 nm). Insets are the schematics of the DW magnetization configuration at each nanowire (top view). (b) Schematic showing the magnetization of the domains at two cross-sections: before and after the DW (c) Plot showing the change in the tilting angle of each DW.

To understand the mechanism of the coupling, the dynamic behaviours of the two DWs are investigated. Shown in Fig. 3 (a) are the magnetization configurations of the two PMA nanowires as functions of their position along the *y* axis (*x* = 5 nm). The applied current density is *J* = 1.2 × 10^12^ A/m^2^. The magnetization configurations of the bottom and upper nanowires are taken at *t* = 25 ns. As compared to the single nanowire system, the magnetizations of the domains are found to be slightly curled towards the *x* axis to minimize the demagnetization energy between the two nanowires (M_x_ ≈ ±3.6 × 10^4^ A/m depending on the position, as will be shown in the cross-sections of the nanowires later). The main difference between the single nanowire system and the two-nanowire system lies in the dynamics of the corresponding DWs. As shown in Fig. 3 (a) and the respective insets (top view), the DWs in the two-nanowire system are aligned along the *y* axis. In other words, the two-nanowire setup has forced the DWs to adopt the Néel configuration. Similar DW configurations have been observed in the work of Bellec *et. al.*^18^. Furthermore, we also see that the two DWs are aligned in the opposite direction, which suggests that they are coupled to each other through their magnetostatic interaction. Fig. 3 (b) shows the cross-section views of the magnetization configuration of the domains. The domains around the DWs are shown to be slightly curled towards the *x* axis (M_x_ ≈ ±3.6 × 10^4^ A/m depending on the position)^14^,^15^. The increased speed of the coupled DWs of the sandwich structure can be attributed to the suppression of Walker breakdown during the DW motions (See Supplementary Information section SI-4)^16^,^19^,^20^. Due to the Néel state of the coupled DWs in sandwich structure (*ψ* = π/2 +*δ*(*t*)), the dynamics can be expressed by:
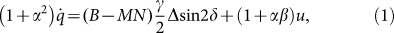

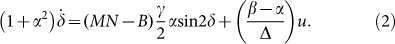
Where *q* is the position of the DW along the nanowire, *γ* is the gyromagnetic constant, *N* is the demagnetization factor of the nanowire, and *ψ* is the tilting angle of the DW in the sandwich structure. *B* is the contribution from the curled magnetic domains (See Supplementary Information section SI-4). It can be seen that the presence of the curled magnetic domains (*B*) suppresses the change in the tilting angle of the DW, which is equivalent to Walker breakdown suppression (See Supplementary Information section SI-4). The *B* constant is approximately equal to 100 mT for the corresponding sandwich structure. Shown in Fig. 3 (c) are magnetization components of the two DWs as a function of the applied current density. The result shows that the magnetizations of the two DWs move closer to the *x* axis for higher current density, which shows that the two DWs gradually change from the Néel configuration to the Bloch configuration as the applied current density is increased. However, we see that the rate at which they rotate is different, which results in weaker coupling as the applied current density is increased. The coupling between the two DWs of the sandwich structure is broken when the upper DW has changed back to the Bloch configuration. The dynamics of the coupled DW for different simulation parameters are discussed in Supplementary Information section S1, S2, S3, S6, and S7.

### Coupled Domain Wall Dynamics in Three-Nanowire System

**Figure 4:**
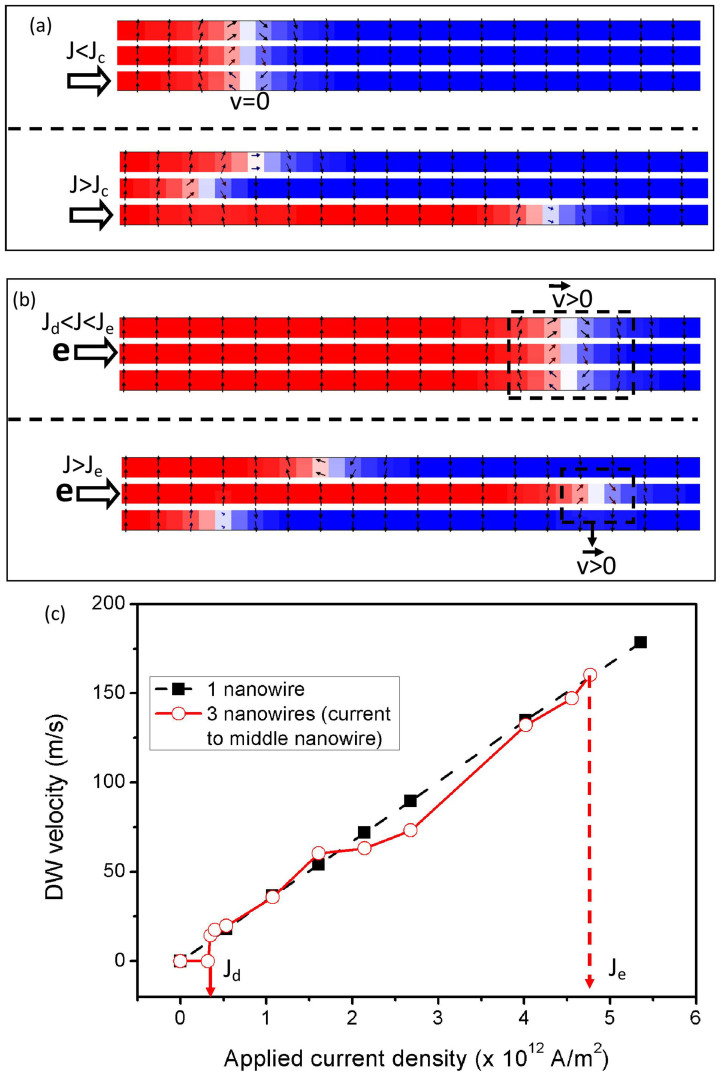
Dynamics of three coupled-PMA DWs of the three nanowire system. (a) Snapshots of the three-nanowire simulations for various range of applied current density (side-view). Current is applied to the bottom nanowire, the coupled DWs are pinned below a critical current density of *J_c_* = 8.03 × 10^12^ A/m^2^. (b) The coupled DWs are able to move together when the current is applied to the middle nanowire and the current density is higher than *J_d_* = 0.4 × 10^12^ A/m^2^ but lower than *J_e_* = 4.8 × 10^12^ A/m^2^. The coupling is broken when the current that is applied to the middle nanowire has exceeded *J_e_*. (c) Plot showing the relation between the speed of the coupled DWs and the applied current density.

We proceed to investigate the coupling and the dynamics of the DWs in a sandwich structure with three PMA nanowires. As compared to the two-nanowire system, the behaviour of the three-nanowire system is determined not only by the applied current density but also by which nanowire the current is applied to. When current is applied to the bottom nanowire, all three DWs of the three nanowires remains stationary (pinned), which is similar to the case of the two-nanowire system when low current density is applied. However, the three DWs remain stationary even under the application of high current density, as shown in the first image of Fig. 4 (a) (side-view). The coupling is only broken when the applied current density is increased beyond *J_c_* = 8.03 × 10^12^ A/m^2^ in which the bottom DW moves independently from the other DWs, as shown in the second image of Fig. 4(a).

When current is applied to the middle nanowire, the behaviour of the three-nanowire system becomes similar to that of the two-nanowire system; i.e. the three DWs are able to move as a group when the applied current density is increased beyond a critical value, as shown in the first image of Fig. 4 (b). The critical current density that is needed to move the three DWs is *J_d_* = 0.4 × 10^12^ A/m^2^, which is lower than the minimum current density that is needed to move the two-DW group (*J_a_*). During the motion, the three DWs move at a speed that is comparable to a single DW in a single nanowire system, as shown in Fig. 4 (c). This result can be attributed to the uncurled domain of the middle nanowire, as explained later. The coupling between the three DWs is again broken when the applied current density is increased beyond *J_e_* = 4.8 × 10^12^ A/m^2^, which allows the middle DW to move independently from the other DWs, as shown in the second image of Fig. 4 (b).

**Figure 5:**
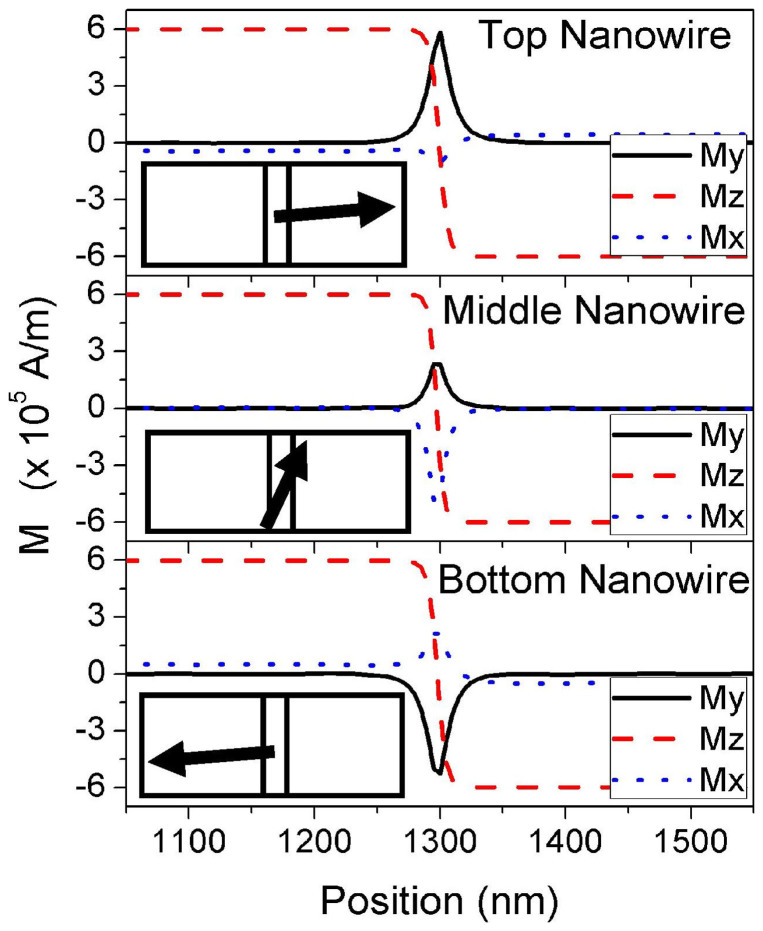
Magnetization configuration of the three-nanowire system. The magnetization configuration of the three-nanowire system (left-edge of nanowire, x = 5 nm). Inset are the schematics of the DW magnetization configuration at each nanowire (top view).

**Figure 6:**
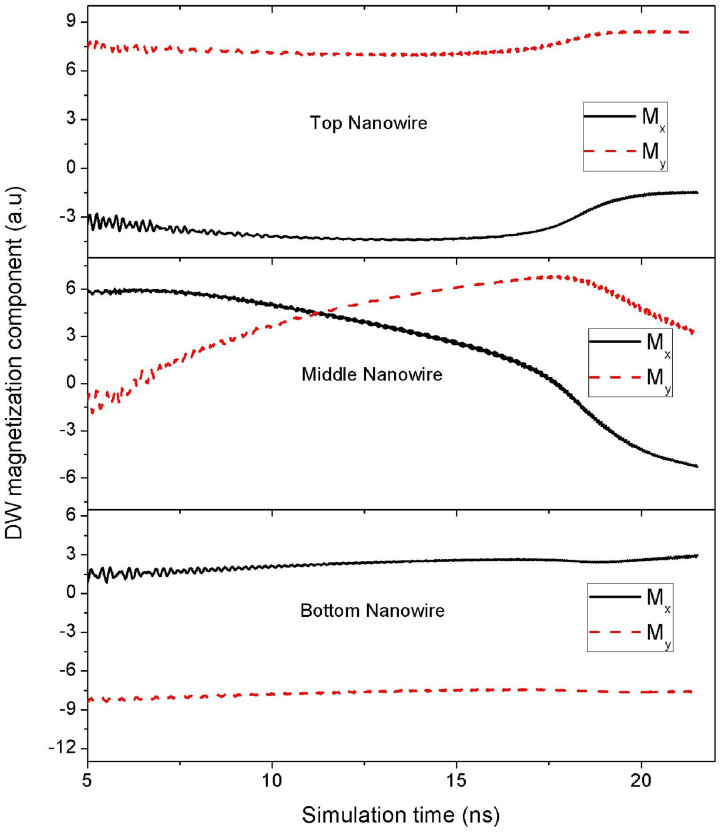
Magnetization dynamics of the three-nanowire system. Plots showing the dynamics of the components of the DWs in the three-nanowire sandwich structure.

The magnetization configurations of all three nanowires are shown in Fig. 5 (left-edge of nanowire, x = 5 nm). Current is applied at the middle nanowire, and the magnetization configurations are taken at *t* = 20 ns after the start of the simulation. The applied current density is *J* = 2.68 × 10^12^ A/m^2^. The DW in the middle nanowire behaves similarly to a DW in a single nanowire system; as shown in the corresponding inset of Fig. 5 (top view). The middle DW is free to rotate along the *xy* plane as it propagates within the middle nanowire. The behaviour of the middle DW can be attributed to the unique magnetization configuration of the middle nanowire. As shown in Fig. 5, the domains in the middle nanowire are aligned perfectly along the perpendicular *z* axis, while the domains of the top and bottom nanowires of the three-nanowire system are slightly curled along the *x* axis, similar to the top and bottom nanowires of the two-nanowire sandwich structure. The magnetization configuration of the middle nanowire then becomes similar to the magnetization configuration of a single nanowire system. This in turn allows the middle DW in the three-nanowire system to rotate in the *xy* plane and achieve a speed that is similar to a DW in a single nanowire system, as shown in Fig. 6.

## Discussion

We have shown that coupled DWs in PMA sandwich structure are able to form the Néel configuration under zero external field or current. When current is applied to one of the nanowires, the coupled DWs of a two-nanowire system are able to move faster than a single DW for a specific range of applied current due to the Walker breakdown suppression. When the stacking of the sandwich structure is increased to include three nanowires, the simulations show that the DWs are still coupled to each other and they are still able to move together when current is applied to the middle nanowire. However, the magnetic domains of the middle nanowire are not curled anymore, and thus the speed of the coupled DW in the three-nanowire system is similar to that of the single nanowire system. To reduce the operation current, we have found that it can be achieved by reducing the thickness of the sandwich structure, as discussed in the Supplementary Information section SI-7. For instance, the threshold current is reduced to *J* = 1.5 × 10^11^ A/m^2^ when the thickness of both the top and the bottom nanowires of the sandwich structure is reduced to 1 nm. The ability to increase the propagation speed of the PMA DW by making use of the two-nanowire system under the application of current is potentially useful in the design of non-volatile magnetic memory devices, therefore the results that are presented here shall give valuable insights into the DW dynamics in those devices. A possible device structure is discussed in details in the Supplementary Information section SI-5.

## Method

The DW dynamics in the PMA sandwich structure are investigated by using the OOMMF [**OOMMF** by Donahue, M. J. and Porter, D. G., **NISTIR 6376** (Sept 1999)] micromagnetic simulation program, with the addition of the spin-transfer torque term to the Landau-Lifshitz-Gilbert (LLG) equation. Fig. 1 (c) shows the schematic diagram of the simulation model, whereby two to three PMA nanowires were stacked vertically with non-magnetic insulating spacer layers between them. The thickness of all spacer layers was *h = * 2 nm, while the width and the thickness of the PMA nanowires were *w = * 40 nm and *t* = 6 nm, respectively. The material parameters were initially set to: saturation magnetization (*M_s_*) = 6 × 10^5^ A/m, exchange stiffness constant (*A*) = 1.3 × 10^−11^ J/m. The coupled Néel DWs have also been observed with spacer layer thickness up to 5 nm (See Supplementary Information section SI-7). The damping constant (*α*) = 0.01, non-adiabatic spin-transfer constant (*β*) = 0.04, and magnetocrystalline anisotropy (*K*) = 4 × 10^5^ J/m^3^ 7. The chosen mesh size is 5 nm × 5 nm × 2 nm. For the chosen non-adiabatic spin transfer constant, the Walker breakdown limit was found to be in the order of 10^12^ A/m^2^ 7.

## Author Contributions

I.P. designed the simulations, analyzed the results, and wrote the manuscript. I.S.K. and G.J.L. performed the simulations and analyzed the results. The study is supervised by W.S.L. All authors contributed to discussion and preparation of manuscript.

## Supplementary Material

Supplementary Information supplementary information

## References

[b1] Parkin S. S. P., Hayashi M. & Thomas L. Magnetic Domain Wall Racetrack Memory. Science 320, 190 (2008). 18403702 10.1126/science.1145799

[b2] Hayashi M., Thomas L., Moriya R., Rettner C. & Parkin S. S. P. Current-Controlled Magnetic Domain-Wall Nanowire Shift Register. Science 320, 209 (2008). 18403706 10.1126/science.1154587

[b3] Hayashi M., Thomas L., Rettner C., Moriya R. & Parkin S. S. P. Direct observation of the coherent precession of magnetic domain walls propagating along permalloy nanowires. Nat. Phys. 3, 21 (2007).

[b4] Hayashi M. *et al.* Current Driven Domain Wall Velocities Exceeding the Spin Angular Momentum Transfer Rate in Permalloy Nanowires. Phys. Rev. Lett. 98, 037204 (2007). 17358722 10.1103/PhysRevLett.98.037204

[b5] Langner H. H., Bocklage L., Kruger B., Matsuyama T. & Meier G. Magnetic domain-wall depinning with reduced current density by short pulse rise time. Appl. Phys. Lett. 97, 242503 (2010).

[b6] Kläui M. *et al.* Direct Observation of Domain-Wall Configurations Transformed by Spin Currents. Phys. Rev. Lett. 95, 026601 (2005). 16090707 10.1103/PhysRevLett.95.026601

[b7] Fukami S., Suzuki T., Ohsima N., Nagahara K. & Ishiwata N. Micromagnetic analysis of current driven domain wall motion in nanostrips with perpendicular magnetic anisotropy. J. Appl. Phys. 103, 07E718 (2008).

[b8] Berger L. Motion of a magnetic domain wall traversed by fast-rising current pulses. J. Appl. Phys. 71, 2721 (1992).

[b9] Komine T., Ooba A. & Sugita R. Current-induced domain wall motion in a multilayered nanowire for achieving high density bit. J. Appl. Phys. 111, 07D314 (2012).

[b10] Ooba A., Komine T. & Sugita R. Micromagnetic study of novel domain wall motion modes in bilayer nanowire with low saturation magnetization. J. Appl. Phys. 113, 203915 (2013).

[b11] Kondou K. *et al.* Electrical Investigation of Notch Width Dependence of Domain Wall Structure in Co/Ni Nanowires. Jpn. J. Appl. Phys. 50, 073002 (2011).

[b12] Purnama I., Chandra Sekhar M., Goolaup S. & Lew W. S. Current-induced coupled domain wall motions in a two-nanowire system. Appl. Phys. Lett. 99, 152501 (2011).

[b13] Purnama I., Chandra Sekhar M. & Lew W. S. Remote driving of multiple domain walls due to topological interaction. Appl. Phys. Lett. 104, 092414 (2014).

[b14] Chandra Sekhar M. *et al.* Helical domain walls in constricted cylindrical NiFe nanowires. Appl. Phys. Lett. 101, 152406 (2012).

[b15] Piao H.-G., Shim J.-H., Djuhana D. & Kim D.-H. Intrinsic pinning behavior and propagation onset of three-dimensional Bloch-point domain wall in a cylindrical ferromagnetic nanowire. Appl. Phys. Lett. 102, 112405 (2012).

[b16] Lewis E. R. *et al.* Fast domain wall motion in magnetic comb structures. Nat. Mater. 9, 980 (2010). 20890280 10.1038/nmat2857

[b17] Koyama T. *et al.* Wire Width Dependence of Threshold Current Density for Domain Wall Motion in Co/Ni Nanowires. IEEE Trans. Magn, 47, 3089–3091 (2011).

[b18] Bellec A., Rohart S., Labrune M., Miltat J. & Thiaville A. Domain wall structure in magnetic bilayers with perpendicular anisotropy. Euro. Phys. Lett. 91, 17009 (2010).

[b19] Miron I. M. *et al.* Fast current-induced domain-wall motion controlled by the Rashba effect. Nat. Mater 10, 419 (2011). 21572411 10.1038/nmat3020

[b20] Thiaville A., Nakatani Y., Miltat J. & Suzuki Y. Micromagnetic understanding of current-driven domain wall motion in patterned nanowires. Euro. Phys. Lett. 69, 990 (2005).

