# Supplementary material for: Coupled Néel domain wall motion in sandwiched perpendicular magnetic anisotropy nanowires
Source: Sci Rep. 2015 Mar 4;5:8754. doi: 10.1038/srep08754 (PMC4348645; doi:10.1038/srep08754)
Supplement: Supplementary Information [file srep08754-s1.pdf]

Supplementary Information

Coupled Néel domain wall motion in sandwiched perpendicular magnetic anisotropy nanowires

I. Purnama, I. S. Kerk, G. J. Lim, and W.S. Lew*

School of Physical and Mathematical Sciences, Nanyang Technological University

21 Nanyang Link, Singapore 637371

*Corresponding author: wensiang@ntu.edu.sg

This file includes:

Details of coupled DW dynamics and device design SI-1 to SI-8

Supplementary Figures S1 to S13

SI-1) Coupled domain wall (DW) dynamics in two-nanowire sandwich structure as a function of time.

Fig. S1 shows the magnetizations of the upper and bottom nanowires of the sandwich structure with current $J = 2.68 \times 10^{12}$ A/m^2 applied to the bottom nanowire. The magnetizations of the two DWs are shown to gradually change over time from its initial state to the stable state. Fig. 3 (a) and (c) of the manuscript corresponds to the stable states of the two DWs (t = 25 ns).

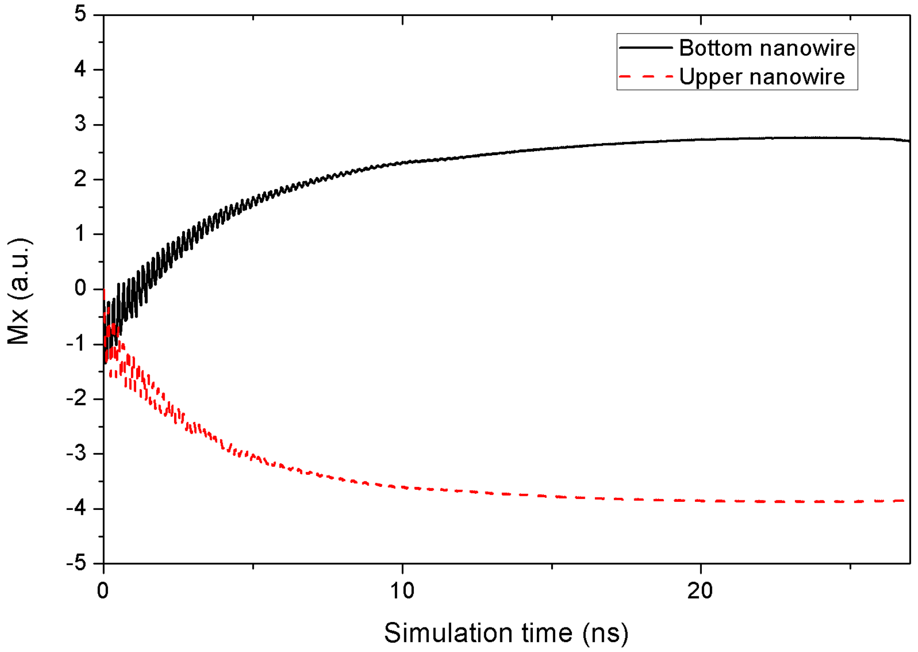

Fig. S1. The magnetization component of the DWs in the bottom and upper nanowires as functions of simulation time.

SI-2) Coupled domain wall (DW) dynamics for different saturation magnetization

We have performed additional simulations to investigate the dynamics of the DWs in the sandwich structure for different saturation magnetization values.

Fig. S2 shows the DW dynamics for various values of saturation magnetization.

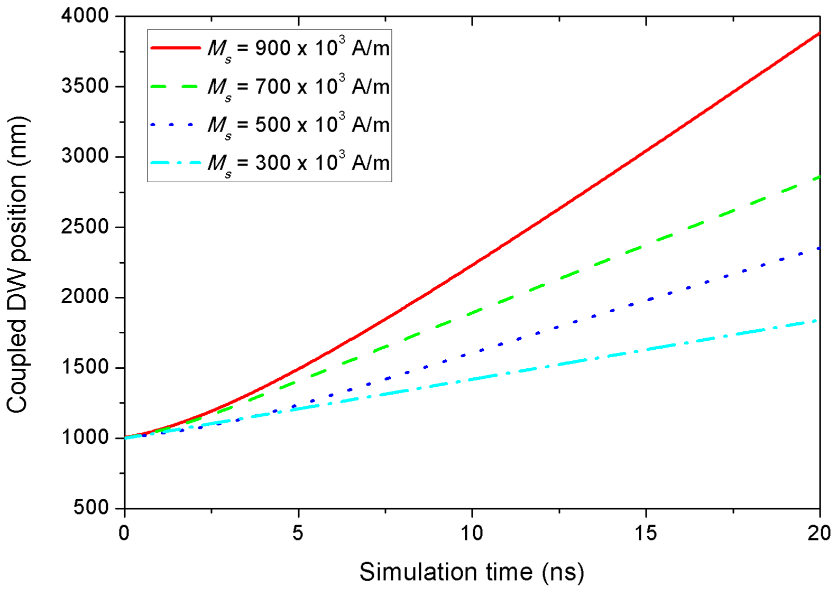

Fig. S2 The coupled DW position as a function of simulation time for different values of saturation magnetization.

The simulation results show that the coupled DW dynamics remain the same even with different values for the saturation magnetization. The change in the value of the saturation magnetization only affects the speed of the coupled DW upon the application of current.

SI-3) Coupled domain wall (DW) dynamics for different values of the spin transfer torque constant (*β*)

We have performed additional simulations to investigate the dynamics of the coupled DWs in the sandwich structure with respect to a change in the non-adiabatic constant. Fig. S3 (a) shows the DW position vs time plots of the coupled DWs in the sandwich structure for various values of the non-adiabatic constant. The speed of the coupled DWs is shown to increase with increasing value for the non-adiabatic spin transfer constant. Fig. S3 (b) shows the corresponding dynamics of the DW in the bottom nanowire. The two graphs show that the change in the nonadiabatic constant of the spin-transfer-torque equation results in the increase of the propagation speed and also in the tilting angle of the DW, which both agree with the 1-D equation for DW dynamics below the Walker breakdown regime.

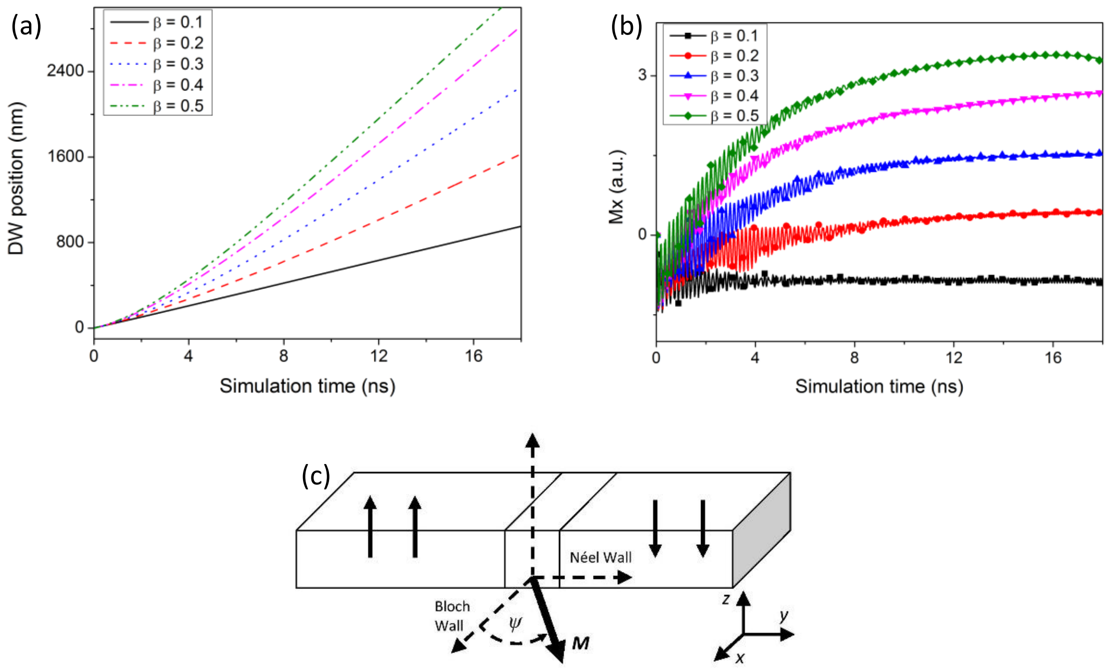

Fig. S3 (a) The speed of the coupled DWs in the sandwich structure for different values of the non- adiabatic spin transfer constant. (b) The magnetization component along the *x* direction of the DW in the bottom nanowire. (c) Schematic of one of the nanowires to illustrate the coordinate axis.

In general, the coupled DWs behave in the same way in all the simulations, i.e. it is driven along the nanowire and tilted in the *xy* plane at the same time. Below the Walker regime, the change in the non-adiabatic constant only results in the change of the propagation speed and the tilting angle. Below are some articles which reported non-zero values for the non-adiabatic constant:

- Koyama, T., Ueda, K., Kim, K.-J., Yoshimura, Y., Chiba, D., Yamada, K., Jamet, J.-P., Mougin, A., Thiaville, A., Mizukami, S., Fukami, S., Ishiwata, N., Nakatani, Y., Kohno, H., Kobayashi, K., and Ono, T., Current-induced magnetic domain wall motion below intrinsic threshold triggered by Walker breakdown, *Nat. Nano.* 7, 635 (2012)
- Burrowes, C., Mihai, A. P., Ravelosona, D., Kim, J.-V. Chappert, C., Vila, L., Marty, A., Samson, Y., Garcia-Sanchez, F., Buda-Prejbeanu, L. D., Tudosa, I., Fullerton, E. E. and Attané, J.-P. Non-adiabatic spin-torques in narrow magnetic domain walls, *Nat. Phys.* 6, 17 (2010)

- Boulle, O., Kimling, J., Warnicke, P., Klaui, M., Rudiger, U., Malinowski, G., Swagten, H. J. M., Koopmans, B., Ulysse C., Faini, G., Nonadiabatic Spin Transfer Torque in High Anisotropy Magnetic Nanowires with Narrow Domain Walls, *Phys. Rev. Lett*., 101, 216601 (2008)
- San Emeterio Alvarez, L., Wang, K.-Y., Lepadatu, S., Landi, S., Bending, S. J., and Marrows, C. H., Spin-Transfer-Torque-Assisted Domain-Wall Creep in a Co/Pt Multilayer Wire, *Phys. Rev. Lett.* 104, 137205 (2010)

SI-4) DW dynamics in the sandwich structure

The analytical explanation of the coupled DW dynamics is as follow:

The 1-D DW dynamics can be written as:

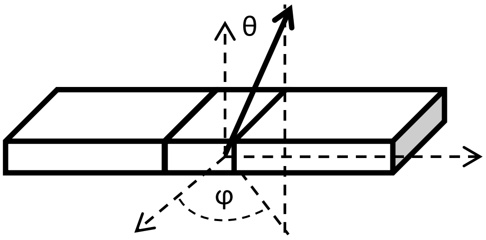

$$\dot{\theta} = -\frac{\gamma}{M_s}\frac{\delta w}{\delta \varphi} - \alpha\dot{\varphi}\sin\theta - u\frac{\delta\theta}{\delta y} - \beta u \sin\theta \frac{\delta\varphi}{\delta y} \ ,$$

$$\sin\theta\dot{\varphi} = \frac{\gamma}{M_s}\frac{\delta w}{\delta\theta} + \alpha\dot{\theta} - u\sin\theta\frac{\delta\varphi}{\delta y} + \beta u\frac{\delta\theta}{\delta y},$$

, where *w* is the volume energy density of the system.

The magnetization of the nanowire can be written as:

$$\theta(y,t) = 2\arctan(\exp[\frac{y-q(t)}{\Delta}]) \ ,$$

$$\varphi(t) = \psi(t) + \gamma(y),$$

$$\gamma(y) = 2C\tan^{-1}(\exp(\frac{(y-q(t))}{\Delta}))$$

, where *q* is the position of the DW along the nanowire, and φ is the tilt angle of the nanowire. The second term in the tilt angle equation is the additional term that comes from the curling domains. *C* is a constant that represents the degree of curling (C<1).

The energy of the system can then be calculated as:

$$w_{EX} = A(\nabla M)^2 = A(\frac{\partial\theta}{\partial y})^2 = A\frac{\sin^2\theta}{\Delta^2} = \frac{A}{\Delta^2}\sec h^2\left(\frac{y-q}{\Delta}\right)$$

$$w_{DEMAG} = \frac{1}{2}H_d \bullet M = \frac{1}{2}M^2 N_z \sin^2\theta\sin^2\varphi \approx \frac{1}{2}M^2 N\sin^2\theta(\sin^2\psi + \sin^2\gamma\cos^2\psi)$$

$$w_{ANI} = K\sin^2\theta = K(\sec h^2\left(\frac{y-q}{\Delta}\right))$$

$$w_{TOT} = w_{DW} = A(\frac{\partial\theta}{\partial y})^2 + K\sin^2\theta + \frac{1}{2}M^2N\sin^2\theta(\sin^2\psi + \sin^2\gamma\cos^2\psi)$$

To obtain the final results, the volume energy density is changed to the areal energy density.

$$\sigma = \int_{-\infty}^{\infty} wdy$$

$$\sigma = \frac{2A}{\Delta} + 2K\Delta + \Delta M^2 N_z(\sin^2\psi + \sin^2\gamma\cos^2\psi)$$

$$B = \frac{MN}{2\Delta}\int_{-\infty}^{\infty}\sin^2\gamma\sin^2\theta dy$$

the derivative of the energy are then

$$\frac{\partial\sigma}{\partial\psi} = \Delta M\sin 2\psi(MN - B)$$

$$\frac{\partial\sigma}{\partial q} = 0$$

The LLG is then rewritten to include the areal energy density:

$$\left(1+\alpha^2\right)\dot{q} = -\frac{\gamma}{2M_s}\left[\alpha\Delta\left(\frac{\partial\sigma}{\partial q}\right) - \left(\frac{\partial\sigma}{\partial\psi}\right)\right] + \left(1+\alpha\beta\right)u$$

$$\left(1+\alpha^2\right)\dot{\psi} = -\frac{\gamma}{2M_s}\left[\left(\frac{\partial\sigma}{\partial q}\right) + \frac{\alpha}{\Delta}\left(\frac{\partial\sigma}{\partial\psi}\right)\right] + \left(\frac{\beta-\alpha}{\Delta}\right)u$$

The dynamics of a current-driven DW in the absence of an applied external field can then be written as:

$$\left(1+\alpha^2\right)\dot{q} = \frac{\gamma}{2}\Delta\sin 2\psi(MN - B) + \left(1+\alpha\beta\right)u$$

$$\left(1+\alpha^2\right)\dot{\psi} = -(MN - B)\frac{\gamma}{2}\alpha\sin 2\psi + \left(\frac{\beta-\alpha}{\Delta}\right)u$$

If we consider the Neel state of the DW in the sandwich structure ($\psi = \pi/2 + \delta$), the dynamics become:

$$\left(1+\alpha^2\right)\dot{q} = (B - MN)\frac{\gamma}{2}\Delta\sin 2\delta + \left(1+\alpha\beta\right)u$$

$$\left(1+\alpha^2\right)\dot{\delta} = (MN - B)\frac{\gamma}{2}\alpha\sin 2\delta + \left(\frac{\beta-\alpha}{\Delta}\right)u$$

It can be seen that the contribution from curling domains (*B*) results in the suppression of the change in the tilt angle of the DW, which is equal to the suppression of Walker Breakdown. It can also be seen that the presence of the curling domains also add to the forward motion of the DW upon the application of current. For sandwich structure with nanowire thickness of 6 nm and width of 40 nm, the contribution from the *B* constant is approximately equal to an anisotropy field of μH ≈ 100 mT.

SI-5) Sandwich structure device details

We have performed additional simulations to investigate the effect of the structure width (*w*) to the dynamics of the coupled DW in the sandwich structure.

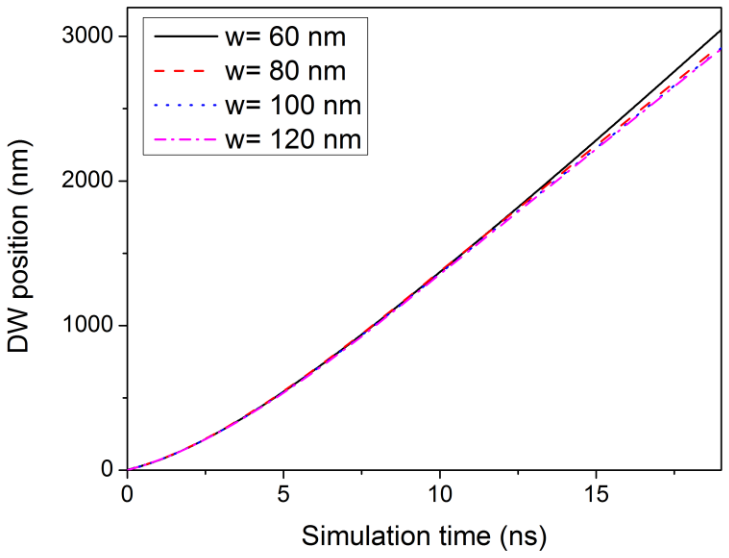

Fig. S4 The coupled DWs position as function of time for different sandwich structure width.

Fig S4 shows that the speed of the coupled DW in the sandwich structure is relatively independent of the width of the structure. To create nanowires with width less than 40 nm, it is advised to do patterning with Electron Beam Lithography (EBL) and ion milling. The techniques are explained in the following report:

- Tavakkoli, K. G, A., Piramanayagam, S. N., Ranjibar, M., Sbiaa, R., Chong, T. C., Path to achieve sub-10-nm half-pitch using electron beam lithography, J. Vac. Sci. Technol., B 29, 011035 (2011).

Below is the schematic of the fabrication process:

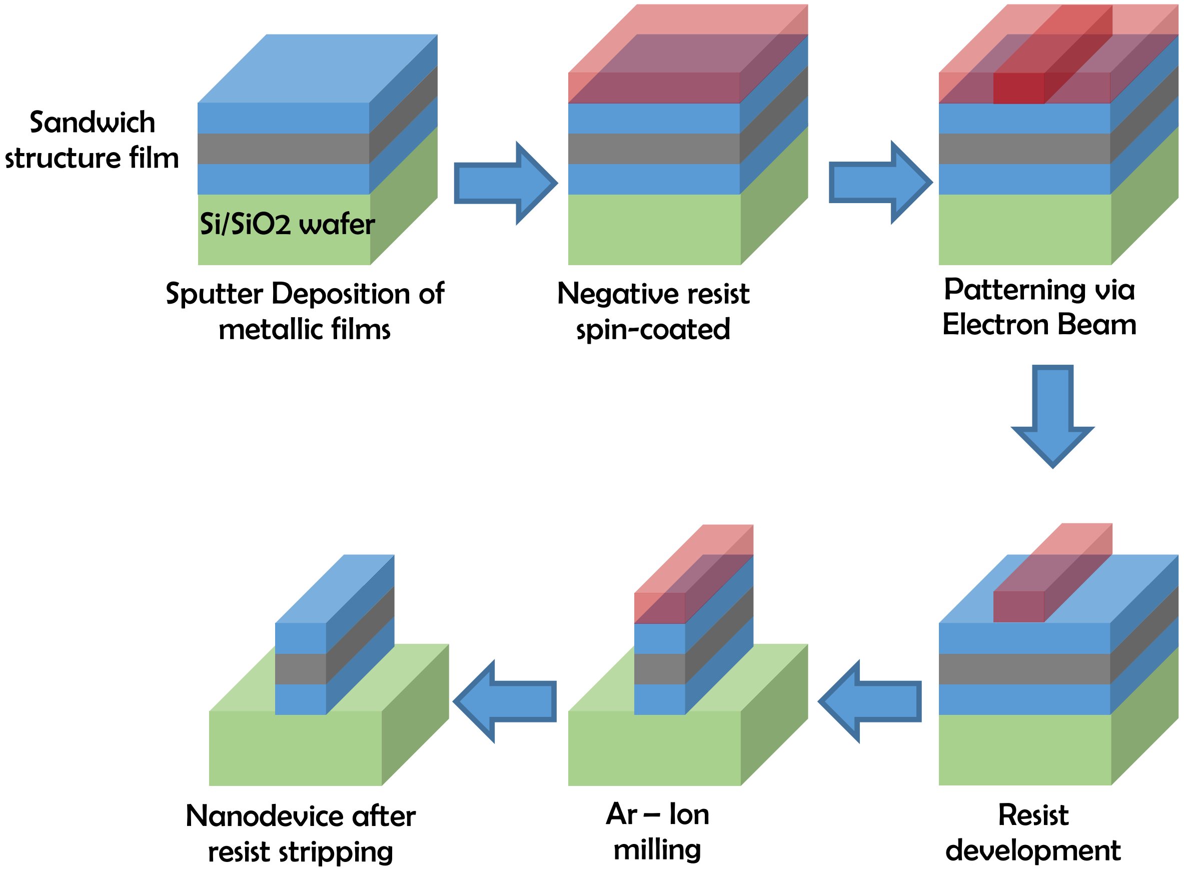

Fig. S5. The fabrication process of the sandwich nanowires.

As shown at Fig. S5, 200nm of negative resist ma-N 2403 (Micro Resist Technology) was spin coated on top of the PMA/insulator/PMA stack. Patterning of the nanowire is done by electron beam lithography with appropriate areal density (~130μC/cm2) and minimal writing current (~15pA) for the best resolution. The unexposed resist was then washed away with developer ma_D 525(Micro Resist Technology). A nanowire pattern with sharp side wall was obtained after argon ion milling and resist stripping processes using removal mr-Rem 400(Micro Resist Technology).

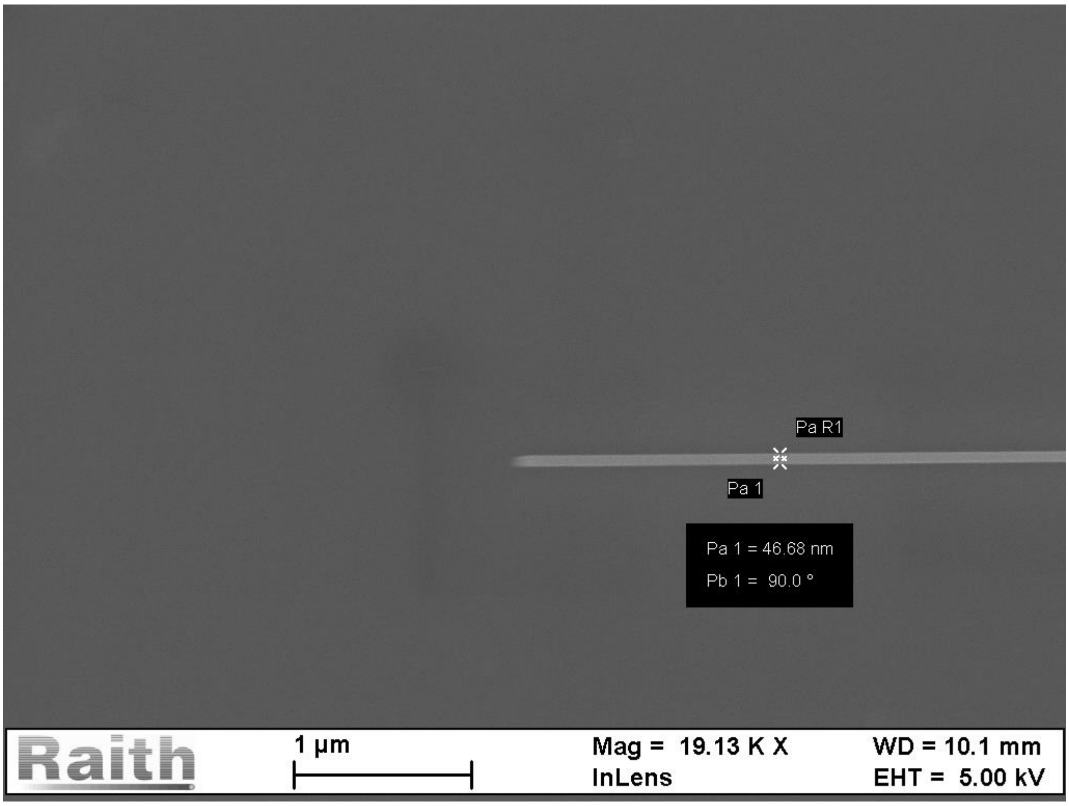

Fig. R2 Image of a 46 nm wide nanowire that is made using the above technique

The gold contact pads can be patterned by using lithography twice: first is for the deposition of the insulator and the second is for the gold contact. The insulator and the gold are patterned in different position so that the gold is able to be connected with the upper nanowire without touching the bottom nanowire.

Fig. S6 shows a schematic of the process:

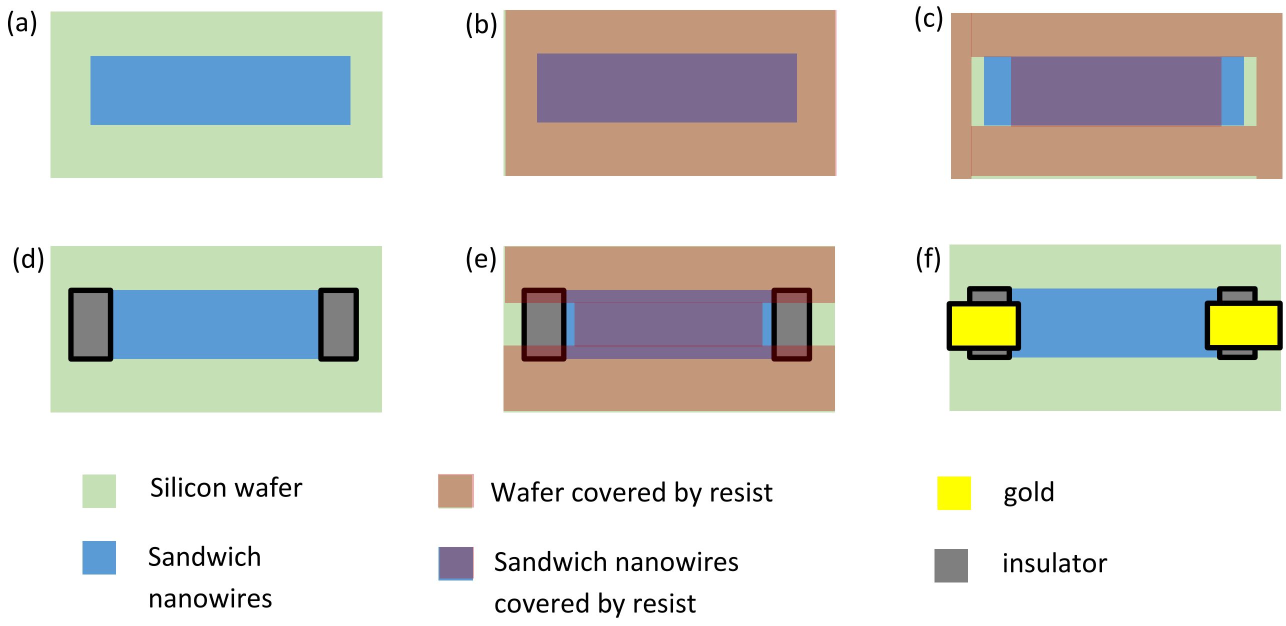

Fig. S6 (a) Initial sandwich structure after the ion milling, top view. (b) Resist deposition (c) Resist after ion milling. Two open areas are created for the insulator deposition. (d) The insulator is deposited and the resist is stripped. (e) Second round of lithography, another pair of open areas is created for the gold deposition. The two areas overlap the insulator to ensure that the gold pads are connected to the top nanowires. (f) Gold pads deposition. (g) Final structure with shortened gold pads for illustration, side view.

The gold pads for the current injection and for the DW injection can follow the common setup for current-driven DW motion experiments. It is possible to modify to the gold pads by extending it to the sides, beyond the insulator, so as to increase the contact area for better control of the device. For the case where three nanowires are involved, an additional lithography and ion milling processes can be employed in the patterning of the sandwich structure.

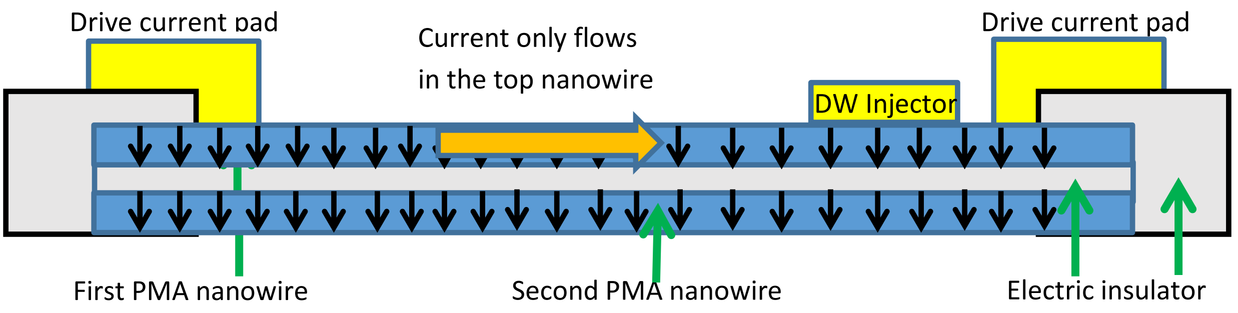

Fig. S7. A design of the DW device with sandwich structure

With the above design, the second PMA nanowires at the bottom are isolated and left as a floating ground. With this design, the current is expected to flow only in the top layer of the sandwich structure, which mimics the results shown in our manuscript. We have also performed additional simulations to investigate how thick the insulator layer at the middle can be made while still maintaining the coupling between the DWs. We found that the DWs can still be coupled with insulator layer of 10 nm, which are thick enough to ensure that there is no current that flows in the lower nanowire.

To generate the coupled DWs, current can be applied to the injection line:

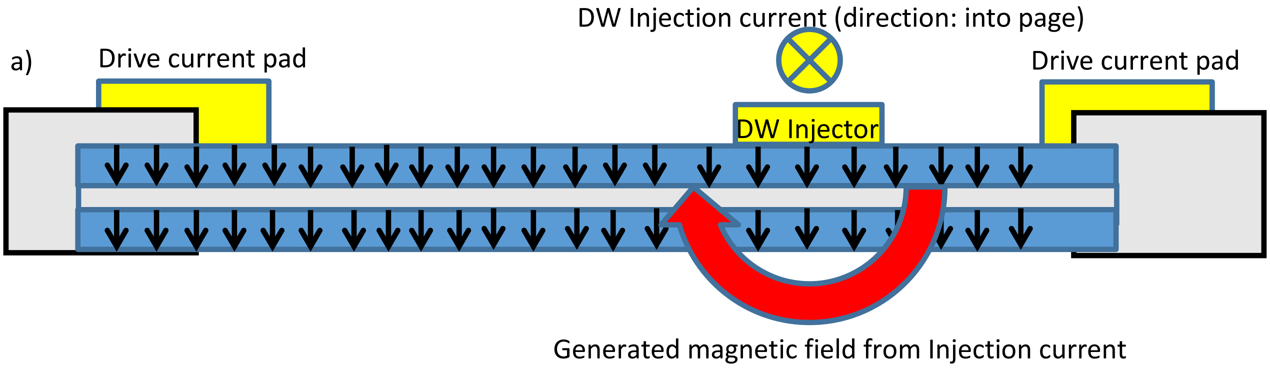

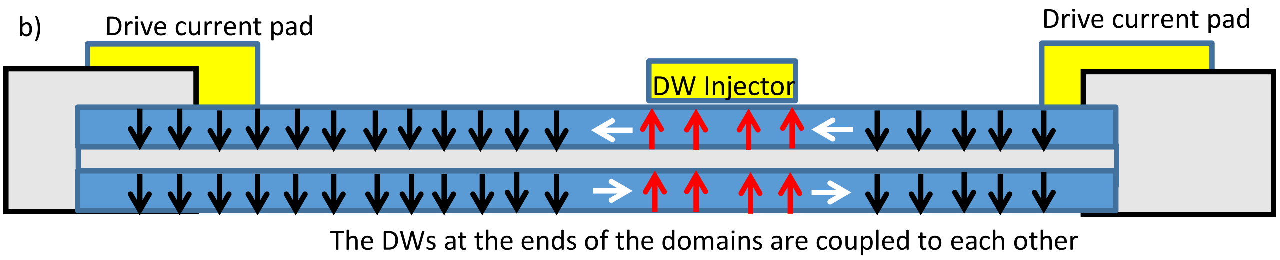

Fig. S8. (sideview) (a) Coupled DW injection process, current is passed through the DW Injection line. A magnetic field is then created around the DW injection line. (b) A new magnetic domain is created in each layers of the sandwich structure due to the magnetic field. The DWs at the sides of the magnetic domains are coupled to each other due to their own stray magnetic fields.

For the three-nanowire sandwich system, the above fabrication method can be extended to add the third nanowire, as shown below:

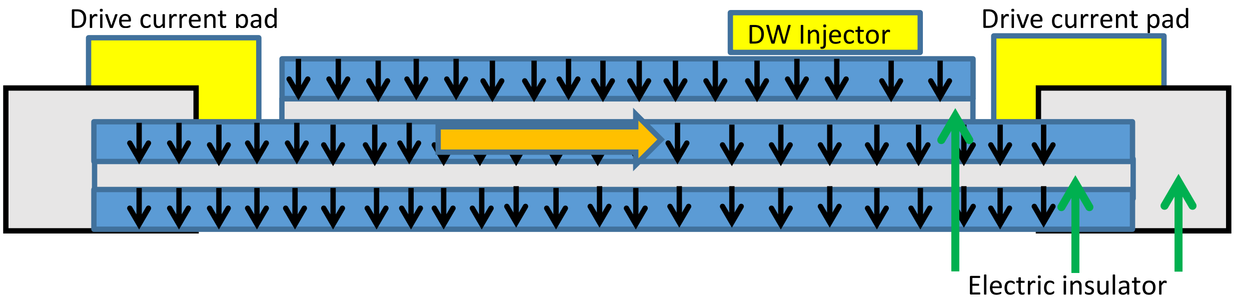

Fig. S9. A design of the three-nanowire structure. The current is expected to flow only in the middle nanowire. The DW injector shall inject DWs into all the nanowires in similar fashion to the two-nanowire structure

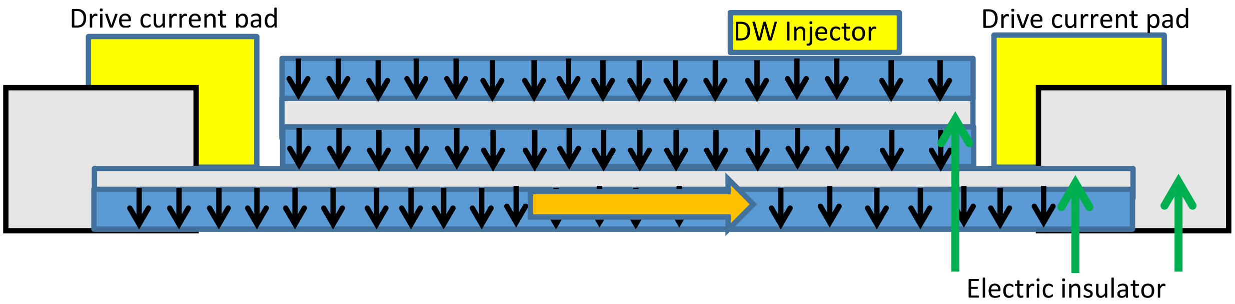

Fig. S10. A design of the three-nanowire structure. The current is expected to flow only in the bottom nanowire. The DW injector shall inject DWs into all the nanowires in similar fashion to the two-nanowire structure

SI-6) DW pinning in sandwich structure

We have performed additional simulations to investigate the effect of extrinsic pinning to the dynamics of the coupled DW in the sandwich structure. Two pinning sites are created in both nanowires of the sandwich structure as shown below in Fig. S11 (a). Current is only applied to the bottom nanowire.

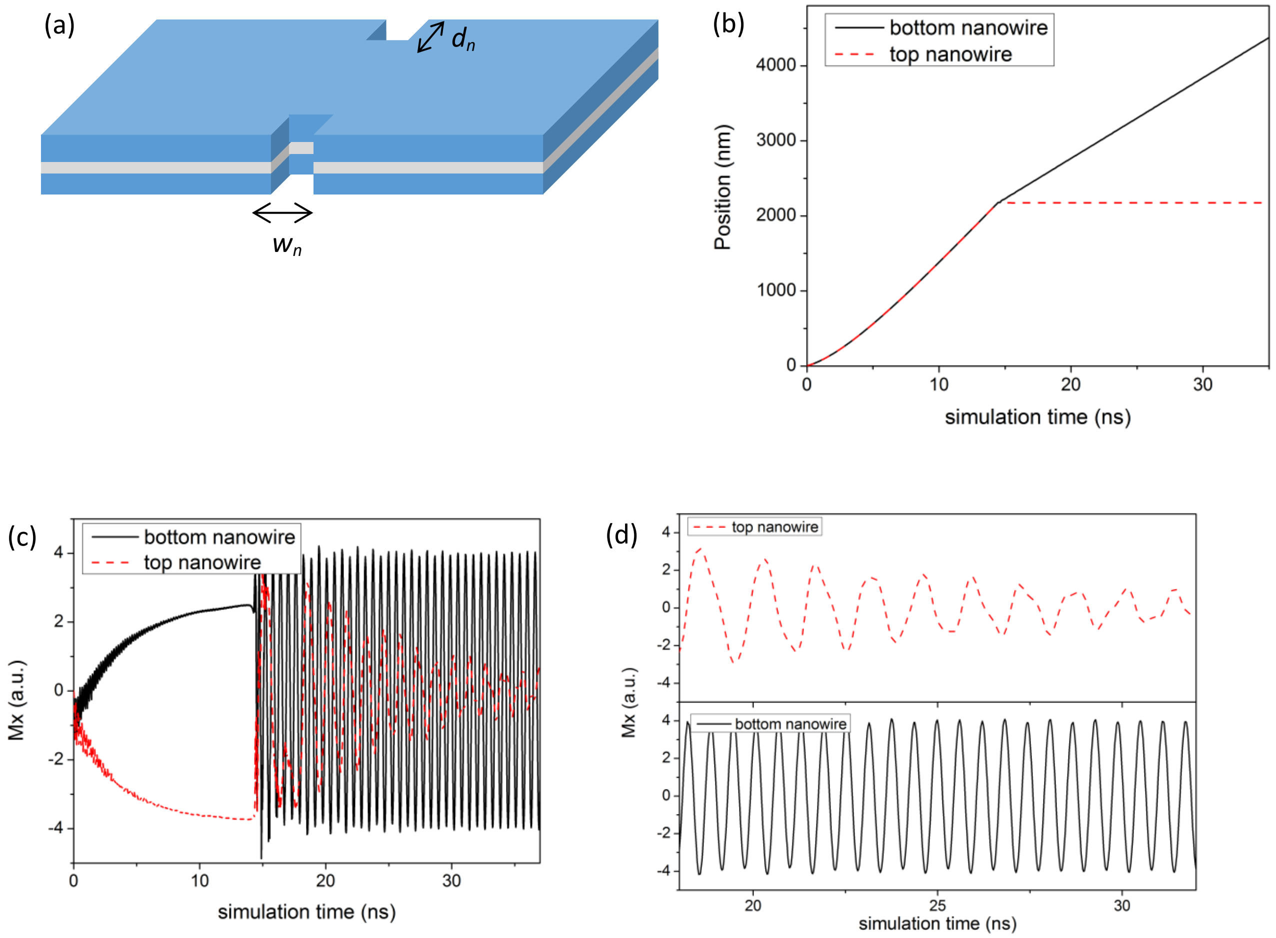

Fig. S11 (a) Schematic of the simulation that is used to investigate the pinning in the sandwich structure. (b) The speed of the DWs in the bottom and the top of the nanowires with pinning sites as functions of time. (c) The magnetization component along the *x* direction of the DWs in the bottom and the top nanowires as functions of time. (d) The magnetization component of the DWs after the coupling breaking.

The width (w_n) and the depth (d_n) of the pinning sites are 10 nm and 10 nm, respectively. The results show that the pinning sites affect the DWs in the top and bottom nanowires individually. Shown in above in Fig S11 (b) are the dynamics of the two DWs when a J = 2.68 $\times 10^{12}$ A/m2 current is applied to the bottom nanowire. Initially the two DWs

are able to move together with nearly a constant speed and a constant tilting angle up to t = 15 ns where they finally arrive at the pinning sites. The DW in the top nanowire is shown to be stopped by the pinning sites, while the DW in the bottom nanowire is shown to be able to continue its movement. Fig. S11 (c) shows that following the coupling breaking, the DWs start to change their behaviour. Fig S11 (d) shows that the DW in the top nanowire starts to come to equilibrium as it oscillates around the x axis with the natural frequency of the spin precession. At the same time, the bottom nanowire starts to rotate in the xy plane which signals that the DW has experienced the Walker breakdown due to the high applied current density.

SI-7) Threshold current density as a function of nanowire thickness

The current that is needed to drive the coupled DWs in the sandwich structure depends on the local pinning that is induced by the curling magnetic domains. We have performed additional simulations with different PMA nanowire thickness to investigate the effect of the structure dimension to the threshold current. Fig. S12 shows the change in the threshold current in sandwich structure with two nanowires with respect to the nanowire thickness. The results show that it is possible to lower the threshold current by making the nanowire thinner. For instance, the threshold current is reduced to J = 1.5 $\times 10^{11}$ A/m^2 when the thickness of both the top and the bottom nanowires of the sandwich structure is reduced to 1 nm.

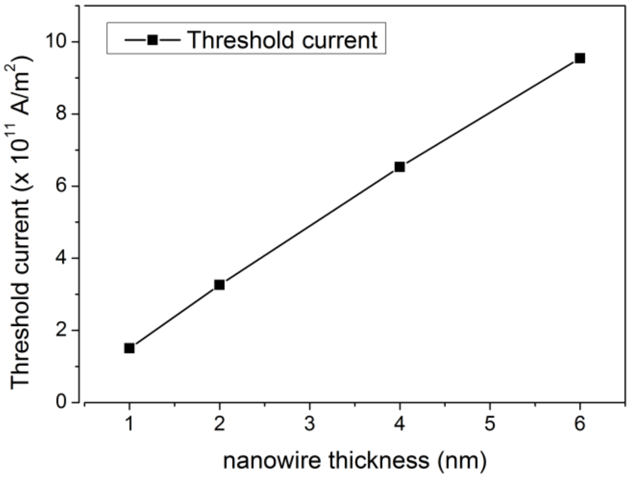

Fig. S12. Threshold current as a function of nanowire thickness. The spacer layer is maintained at 2 nm.

SI-8) Current density distribution within the sandwich structure

We have performed additional simulations using COMSOL to simulate the current density distribution within the sandwich structure. Fig. S13 shows the simulation result. The thickness of each ferromagnetic layer here is 5 nm while the thickness of the middle insulator spacer layer is 2.5 nm. The two smaller boxes at the top of the sandwich structure correspond to the contact pads.

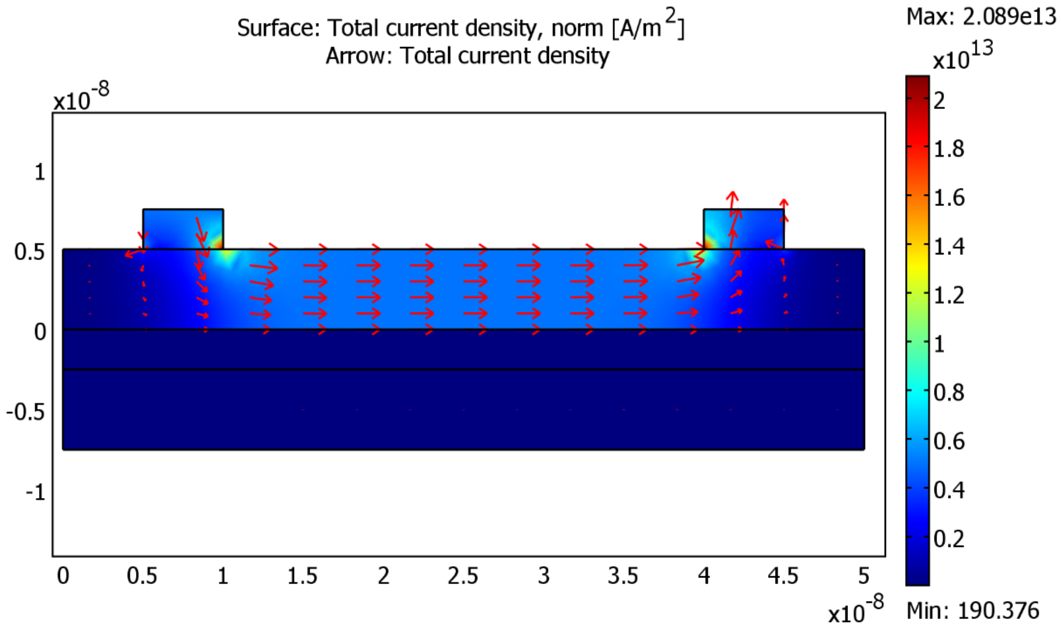

Fig. S13. Current density distribution in the sandwich structure.

The result shows that majority of the flows in the in-plane direction within the top nanowire, with minimal current flowing in the bottom nanowire. Additionally, it is also possible to increase the spacer layer thickness up to 5 nm to ensure even better current isolation while still maintaining the coupling between the DWs in the top and bottom nanowire. At such thickness, it is highly improbable for any current to tunnel through. Therefore we believe that

the current in the sandwich structure can be confined to just flow in one of the nanowires to give the same DW dynamics as discussed in the manuscript.
